# Fluorescence polarization measures energy funneling in single light-harvesting antennas—LH2 vs conjugated polymers

**DOI:** 10.1038/srep15080

**Published:** 2015-10-19

**Authors:** Rafael Camacho, Sumera Tubasum, June Southall, Richard J. Cogdell, Giuseppe Sforazzini, Harry L. Anderson, Tõnu Pullerits, Ivan G. Scheblykin

**Affiliations:** 1 Chemical Physics, Lund University, PO Box 124, Lund, SE-22100, Sweden; 2 Glasgow Biomedical Research Centre, University of Glasgow, G12 8QQ, United Kingdom; 3 Department of Chemistry, University of Oxford, Mansfield Road, Oxford, OX1 3TA, United Kingdom

## Abstract

Numerous approaches have been proposed to mimic natural photosynthesis using artificial antenna systems, such as conjugated polymers (CPs), dendrimers, and J-aggregates. As a result, there is a need to characterize and compare the excitation energy transfer (EET) properties of various natural and artificial antennas. Here we experimentally show that EET in single antennas can be characterized by 2D polarization imaging using the single funnel approximation. This methodology addresses the ability of an individual antenna to transfer its absorbed energy towards a single pool of emissive states, using a single parameter called energy funneling efficiency (ε). We studied individual peripheral antennas of purple bacteria (LH2) and single CP chains of 20 nm length. As expected from a perfect antenna, LH2s showed funneling efficiencies close to unity. In contrast, CPs showed lower average funneling efficiencies, greatly varying from molecule to molecule. Cyclodextrin insulation of the conjugated backbone improves EET, increasing the fraction of CPs possessing ε = 1. Comparison between LH2s and CPs shows the importance of the protection systems and the protein scaffold of LH2, which keep the chromophores in functional form and at such geometrical arrangement that ensures excellent EET.

Efficient light-harvesting, i.e. absorption of the solar radiation and transport of this energy towards a special site, is crucial for numerous applications that use photons for driving chemical reactions, producing mechanical work or electricity. The best example of this is natural photosynthesis where the absorbed energy flows from one antenna system to another, ending up in so-called reaction centers (RC)^1^,^2^.

There are numerous literature studies reporting very efficient transport of excited states in natural antennas, such as the peripheral antenna complex of purple bacteria LH2^3^,^4^,^5^,^6^,^7^,^8^, chlorosomes^9^,^10^ and others^1^. In the light-harvesting machinery, the energy flow can be studied in great details using time-resolved and high resolution spectroscopy due to the energetic differences between donor and acceptor states^3^,^4^,^5^,^6^,^7^,^8^,^9^,^10^,^11^,^12^. Basically, in such experiments spectral differences between donor states and acceptor states are used to monitor the energy transfer. For some light-harvesting antennas, such as LH2, the spectroscopic data can be related to the spatial location of the excitations at a given time after the light absorption. This is due to the detailed knowledge about the antennas’ structure obtained through crystallography methods. Moreover, having detailed structural information together with time-resolved dynamics allows for sophisticated theoretical modeling of the excitation energy transfer (EET) in these systems^2^,^13^,^14^.

However, assessing EET by the aforementioned methods becomes much more difficult when the states involved are spectroscopically similar (homo-EET between e.g. individual states in a single B850 ring of LH2), particularly at room temperature. This is because spectral differences between donor and acceptor states, if any, are very small. On the other hand, in such conditions EET can be detected by either fluorescence anisotropy measurements^5^,^6^,^9^,^11^,^12^ or by exciton-exciton annihilation studies^15^,^16^,^17^. Again, these methods greatly benefit from knowing the structure and the size of the antennas, while becoming much less informative if these parameters are not known.

Currently, much research is focused on mimicking natural photosynthesis and natural antennas by creating artificial light-harvesting systems, for example, using ensembles of organic dyes, J-aggregates, conjugated polymers and different types of nanoparticles^18^,^19^,^20^,^21^,^22^,^23^. As a result, there is a need to characterize the EET properties of various molecular ensembles. Contrary to natural antenna systems where the number of chromophores and their spatial arrangement is determined in great details, structure and even size of artificial antennas is usually not well defined or only limited information is available. Therefore, the community would greatly benefit from a method to assess the EET efficiency in multichromophoric antenna systems which does not require knowledge of the system structure, as an alternative and/or complementary approach to time resolved measurements and theoretical modeling based on detailed structural information.

Such method should concentrate on the result of the EET, basically on the location of the excitation after complete relaxation, and not on the fact of EET between chromophores as such or on energy transfer rates. This is because we are interested in monitoring and quantitatively characterizing the so-called energy funneling: the ability of an individual antenna to transfer all of its absorbed energy to a single common pool of states. This terminology has been widely used when talking about artificial antennas, starting from the pioneering work on single molecule spectroscopy (SMS) of conjugated polymers (CPs)^24^,^25^.

Single-molecule methods allow detecting the fluorescence of individual molecules, giving information beyond the ensemble average. SMS has been used to study EET in multichromophoric antennas^2^,^24^,^25^,^26^,^27^,^28^,^29^,^30^,^31^,^32^,^33^,^34^ Besides fluorescence light polarization, which will be discussed below, it is the collective fluorescence quenching (blinking) that is commonly used to detect EET^24^,^25^,^31^,^34^. The collective quenching of a large number of chromophores in a single antenna can only be explained by efficient energy migration towards a photo-generated quenching site. However, it remains unknown how the energy migration occurs in the undisturbed antenna, i.e. before the photo-generated quencher altered the antenna’s energy landscape.

Therefore, one would like to have a method that is not based on fluorescence blinking (quenching) to answer the following two questions: (1) can the excitations travel through the antenna irrespectively of their initial location? and (2) is the transfer directional (the energy goes towards a specific place or states) or random? The answer to these questions should come in the form of a quantitative parameter that allows for comparing different antennas, potentially made of different materials and having different conformations.

The theoretical ground for a method able to answer these questions was recently reported by some of us^30^. This analysis procedure receives the name of single funnel approximation (SFA) and is based on a polarization-sensitive single molecule experiment called 2D polarization imaging (2D-POLIM)^27^. 2D-POLIM allows to correlate the polarization properties in fluorescence excitation and emission. Using the SFA, the light-harvesting of a single antenna is measured through a parameter ε - energy funneling efficiency, which tells how close the observed optical properties of an antenna are to that of a ‘perfect antenna’. For example, if the antenna’s fluorescence always comes from the same pool of emitting states independently on the way the antenna is excited, then this antenna has 100% funneling efficiency, corresponding to ε = 1. In other words, in a perfect antenna, the excitation energy regardless of its initial location always migrates (funnels) to the same pool of emitting sites or ‘tip of the funnel’.

In the current contribution we test this method experimentally on two well-known multichromophoric systems (Fig. 1): single LH2 complexes – the famous natural antenna of purple bacteria, and single molecules of conjugated polymers (CPs, polyfluorene bis-vinylphenylene derivatives). The purpose of the study is twofold: (i) to see if the SFA measures high funneling efficiencies in LH2, which the scientific community knows as a perfect antenna, and (ii) to check whether a CP chain has a chance to compete with natural antenna systems. The applicability of 2D POLIM to assess the quality of individual light harvesting systems is supported by the very large energy funneling efficiencies obtained for LH2 complexes, as expected from the whole body of theoretical end experimental investigations of LH2. Further, the results demonstrate that despite the potential for efficient energy funneling in CPs, improvements towards a robust structural stability at an optimal configuration are needed to achieve the efficiency of natural antennas.

## Two dimensional polarization imaging

In 2D POLIM a single object is excited by linearly polarized light and its fluorescence detected through an emission analyzer (polarizer, see Experimental setup and Supplementary information for more details). This way the fluorescence intensity *I* of the object is recorded as a function of both the excitation polarization plane orientation (*φ*_*ex*_) and the orientation of the emission analyzer (*φ*_*em*_).

This two dimensional function *I* (*φ*_*ex*_ , *φ*_*em*_) is called polarization portrait^27^,^30^. As described in the following paragraphs, the polarization portrait of a single system reflects the internal organization of its chromophores and the EET occurring among these chromophores (intra-EET).

## General theory. 

Let us consider the response of a multichromophoric system, composed of N chromophores (dipoles). To simplify our discussion, let us assume that in each chromophore the transition dipole moments for fluorescence excitation and emission are collinear. Thus, the function *I* (*φ*_*ex*_ , *φ*_*em*_) can be expressed as:


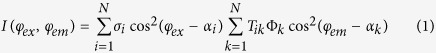


where σ_i_ and α_i_ are the absorption cross section and in-plane orientation of the dipole *i*, respectively. Due to EET, the excited state initially created in dipole *i* can lead to the emission of a photon by a different dipole *k*. The dipole *k* has fluorescence quantum yield Φ_k_ and in plane orientation α_k_. *T*_ik_ is the transfer matrix, where each element of the matrix has values between 0 and 1, and designates the time averaged probability of an excited state created at chromophore *i* to end up in chromophore *k* due to EET. Note that the transfer matrix describes the EET process regardless of its nature (FRET or coherent wave-like motion).

Integration of the polarization portrait over one of the angles, *φ*_*ex*_ or *φ*_*em*_, leads to a cosine-like function (Malus’s law) of the form: *I*_*0*_(1 + *M* cos[2 (*φ* − *θ*) ])^27^. Where, *M* is the modulation depth and *θ* is the phase. *M* describes the degree of alignment of the transition dipole moments, and *θ* represents the main orientation axis. If the polarization portrait is integrated over the emission polarization angle *φ*_*em*_ the modulation depth and the phase reflect the polarization properties of the dipoles responsible for fluorescence excitation: *M*_*ex*_ and, *θ*_*ex*_. On the other hand, when the polarization portrait is integrated over the excitation angle *φ*_*ex*_ the modulation depth and phase reflect the polarization properties of the light emitting dipoles: *M*_*em*_, and *θ*_*em*_. Modulation depths and phases are parameters widely used in single molecule spectroscopy not only for obtaining structural information about the molecules but also for detecting EET^27^,^28^,^29^,^30^,^31^,^32^,^35^,^36^,^37^,^38^,^39^,^40^,^41^.

However, analyzing the full polarization portrait one can obtain quantitative information about EET that is inaccessible via the integrated parameters like polarization modulation depths and phases. How detailed this information about EET is, depends on the knowledge about the spatial arrangement (geometry) of the chromophores in the system. For example, for an antenna containing three chromophores of known orientation a complete picture about the relative EET rates between them can be obtained^27^. If nothing is known about the geometry and number of chromophores, then a phenomenological approach has to be used. So far two methods have been proposed: (i) one based in the average rotation angle between the excited and emissive states^27^, and (ii) another based in a so-called single funnel approximation (SFA)^30^. The latter will be used in this study.

## The single funnel approximation. 

The SFA is a simple and robust methodology to characterize the light harvesting properties of individual antennas. The principle behind the SFA is to express the polarization portrait as a linear combination of two components having a clear physical meaning: *A*—no energy transfer, and *B*—energy transfer. In general terms, the diagonal elements (*k* = *i*) of the transfer matrix *T*_ik_ describe the no energy transfer component *A*, while the off-diagonal elements describe the energy transfer component *B*.

In order to use this representation when the number, orientation, strength and fluorescence quantum yield of the chromophores are not known several approximations have to be made. These approximations are: (i) the EET drives the excitations towards a fixed set of chromophores called EET-emitter (a common pool of emitting states), (ii) the unknown N-dipole system is represented by a new group of 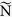 standard dipoles with equal fluorescence excitation cross sections (σ^ex^ ~ σ × Φ), and (iii) each dipole transfers in average the same amount of energy towards the single EET-emitter (see Supplementary Information for more details).

In other words, the SFA assumes that each chromophore transfers *ε* of the total excitations to the EET-emitter while 1- *ε* remains at the initially excited chromophore (Fig. 2). The parameter *ε* is then called *energy funneling efficiency*^30^


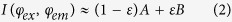



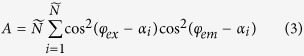



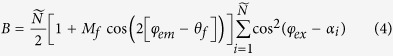


where *A* and *B* describe the behavior of an independent group of chromophores and a single EET-emitter (energy funnel), respectively. For details about the fitting procedure of experimental data using equations 2, 3, 4 see Supplementary Information and ref. 30.

The EET-emitter is the energy acceptor of the multichromophoric system or the “tip” of the energy funnel, and is coupled to all absorbing states, directly or indirectly, by EET processes. It is very important to note that the EET-emitter can consist of more than one dipole transition. Therefore, the EET-emitter can possess any polarization degree (*M*_f_) from dipolar, *M*_f_ = 1, to isotropic, *M*_f_ = 0, while its main orientation is given by *θ*_f_.

For example, if a fraction *ε* of the absorbed energy is always transferred to a single chromophore, then this chromophore represents the common pool of emitting states, which has a dipolar emission. On the other hand, if the absorbed energy is transferred to a group of strongly coupled states that freely exchange excitation energy and thus fluorescence can be emitted from any one of them, then this group of coupled states represents the common pool of emitting states. In this case the polarization degree of the EET-emitter depends on the relative orientation of the emissive transition dipole moments that constitute the pool of emitting states.

## Excitation energy transfer sensitivity. 

In order to illustrate the sensitivity of the SFA to EET processes, Fig. 3 shows qualitatively the behavior of *M*_*em*_ and *ε* as a function of the degree of alignment (ranging from 0 to 0.9 in the units of *M*_*ex*_) and the Förster-like EET rate, for a large ensemble of identical dipoles obtained by a computer simulation^42^. In the absence of EET, *ε* = 0 and *M*_*em*_ = *M*_*ex*_ because the excited and emitting chromophores are the same (independent chromophores). If EET occurs among the chromophores, then *M*_*ex*_ is not affected, *M*_*em*_ slightly increases relative to *M*_*ex*_ because of preferential energy migration towards chromophores oriented close to the main orientation axis^42^, and *ε* increases towards one, being nearly independent on the degree of alignment. This trend for a large ensemble of dipoles can be seen as the average behavior expected for single multichromophoric systems. Note that *M*_*em*_ > *M*_*ex*_ has been experimentally reported for many isolated multichromophoric systems^27^,^28^,^29^,^31^,^32^,^35^,^36^,^37^,^38^,^39^.

## Results

To visualize the extent of EET in isolated PFBV, PFBV-Rtx and LH2 we will use the modulation depth correlation plots, *M*_*ex*_ vs. *M*_*em*_^37^,^39^, and the histograms of funneling efficiency *ε* (Fig. 4). The LH2 complexes were investigated in two separate experiments with excitation wavelength at 800 nm and 850 nm, respectively. The funneling efficiency histograms presented in Fig. 4 corresponds to those single molecules/complexes which polarization portraits could be successfully fitted by the SFA: 94% of the 181 isolated PFBV molecules, 85% of the 141 isolated PFBV-Rtx molecules, 91% of the 545 isolated LH2 complexes excited through the B800 ring, and 92% of the 410 isolated LH2 complexes excited through the B850 ring.

The small fraction of single molecules/complexes that could not be fitted were characterized by very low signal-to-noise ratios and/or unstable polarization properties. Note that antennas possessing strong blinking and photo bleaching cannot be analyzed because no polarization portrait can be constructed under these conditions. On the other hand, it is also possible that a single multichromophoric system cannot be fitted due to its multiple funnel behavior, for example containing two distinct domains of quite different configuration and EET properties. This occurred for few of the isolated PFBV and PFBV-Rtx chains.

In a modulation correlation plot, the larger the spread of the points from the diagonal the larger the extent of EET between differently oriented chromophores^27^,^31^. Particularly, points above the diagonal, the most common feature in our data (Fig. 4A-D), are the result of EET towards a pool of emissive states that are more aligned than those initially excited. Further, the *M*_*ex*_ distributions can be used to obtain information about the geometrical arrangement of the dipoles in the system and thus the structure of the multichromophoric system. In this regard, interpretation of the modulation correlation plots (Fig. 4A–D) have been extensively discussed in our previous works^37^,^39^.

By looking at the modulation correlation plots and the histograms of *ε* (Fig. 4), it is clear that each system is different in terms of their conformation (*M*_*ex*_) and EET properties. The *ε* histograms show that the LH2 complex is by far the best light harvesting antenna, and insulation of the PFBV-Rtx conjugated backbone seems to improve energy funneling compared to the unprotected PFBV.

## Discussion

### Natural light-harvesting antennas—LH2

Any method designed to quantify the quality of a light-harvesting antenna should yield large efficiencies for LH2 complexes. This is because LH2 is a benchmark for light-harvesting as it possesses the photophysical properties desired of an antenna to be used in light-conversion devices. Indeed, 2D-POLIM in combination with the SFA showed *ε* ≈ 1 for individual LH2 complexes justifying the practical use of the method (Fig. 4G,H).

**Figure 1:**
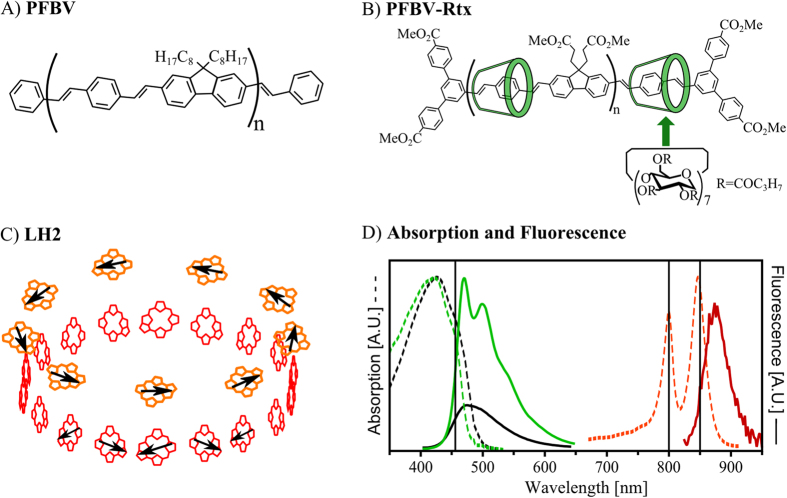
Artificial and natural light-harvesting antennas under study. Chemical structures of (**A**) PFBV (n ~ 10), (**B**) PFBV-Rtx (n ~ 10)^51^,^59^. PFBV-Rtx was obtained by first threading the conjugated moieties into cyclodextrin macrocycles and then polymerizing these units. Therefore, the cyclodextrin rings have no covalent bonds with the conjugated core of PFBV-Rtx. The transition dipole moments of CPs lie preferentially along the direction of the conjugated backbone. This property has been used extensively in single molecule experiments to relate the polarization properties of CPs to their chain conformation^25^,^29^,^31^,^35^,^36^,^37^. (**C**) Spatial configuration of the bacteriochlorophyll a (BChl a) molecules in LH2. The pigments form two concentric rings B800 (orange) and B850 (red) named after their characteristic absorption wavelength. Black arrows illustrate the direction of the transition dipoles in the bacteriochlorophyll molecules. (**D**) Absorption (dashed lines) and emission (solid lines) spectra of PFBV (black), PFBV-Rtx (green) and LH2 (red). Note that only the low-energy part (λ > 700 nm) of the LH2 absorption spectrum is shown for clarity. Further, the fluorescence of PFBV has been scaled relative to PFBV-Rtx to illustrate the larger brightness of PFBV-Rtx molecules. Excitation wavelengths (458, 800 and 850 nm) are indicated by vertical lines.

**Figure 2:**
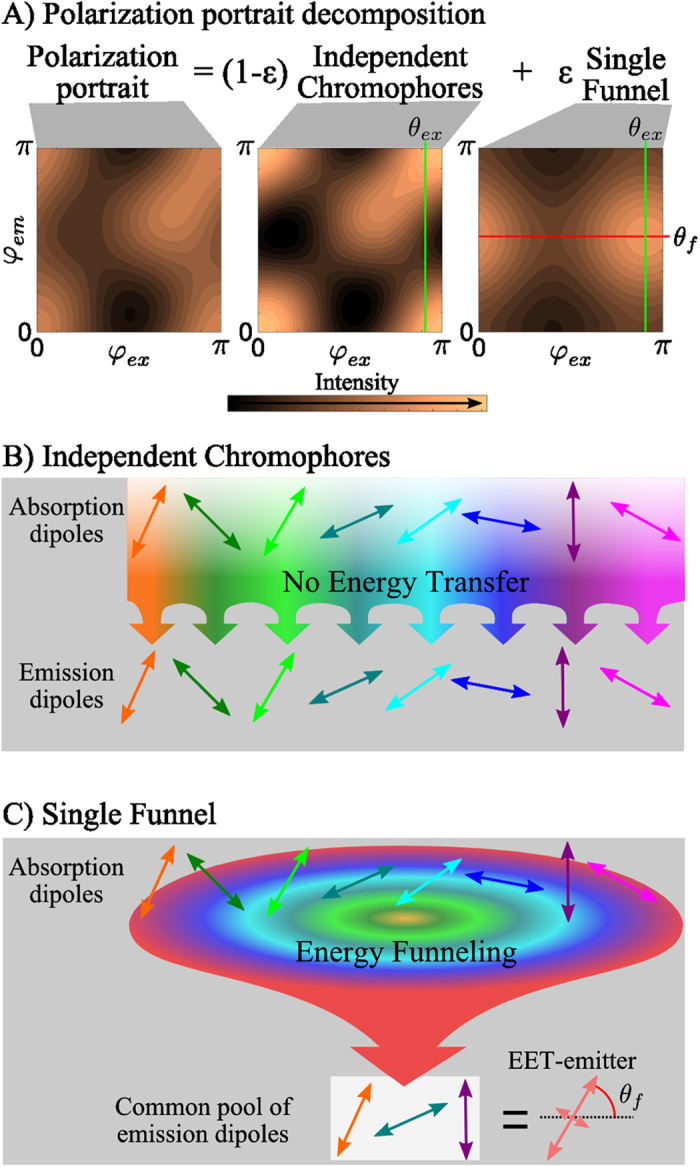
Energy funneling efficiency (ε) determination in the single funnel approximation. (**A**) Representative example of a polarization portrait decomposition. The polarization portrait is presented as a linear combination of two components: ‘Independent Chromophores’ and ‘Single Funnel’. In the former (**B**) the excited and emitting chromophores are the same, while in the latter (**C**) the absorbing states transfer their energy to a single pool of emissive states, (EET-emitter), which emits fluorescence. The parameter ε determines the contribution of the ‘Single Funnel’ component to the whole signal. *θ*_*ex*_ is the main orientation of the dipoles responsible for fluorescence excitation. *θ*_*f*_ is the main orientation of the EET-emitter.

**Figure 3:**
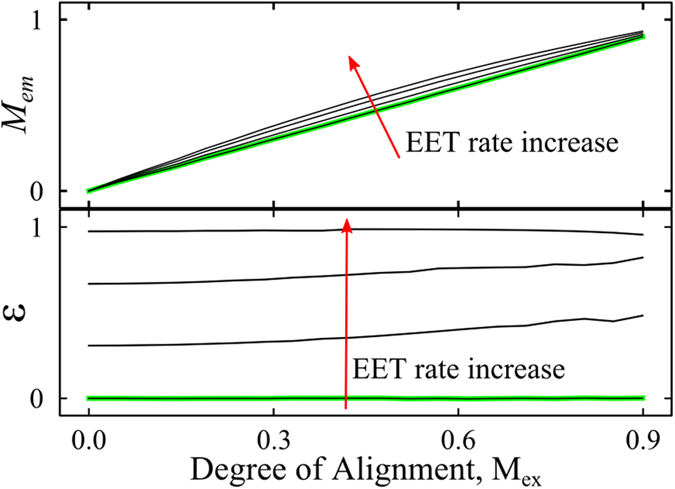
2D-POLIM parameters M_em_ and ε as a function of the degree of alignment and Förster-like EET transfer rate for a large ensemble of identical dipoles^42^. The degree of alignment, which is dependent on the angular distribution of the dipoles, is expressed in the units of *M*_ex_ for simplicity. Green curves were calculated in the absence of EET.

Let us discuss in more details the EET processes occurring within single LH2 complexes, which we probe in two separate experiments using excitation wavelength 800 nm or 850 nm. The LH2 complex has a hollow cylinder structure determined by X-ray crystallography (C9 symmetry, Fig. 1)^43^. The pigments involved in the light capture are the bacteriochlorophylls a (*BChl a*). They are organized into two coaxial rings in the protein scaffold. These two rings are known as B800 (9 *BChl*) and B850 (18 *BChl*) named by their Qy absorption maximum (Fig. 1). The *BChl* molecules in the B800 ring are well-separated (≈20 Å), and thus each pigment can be seen as an individual chromophore. On the other hand, the distance and orientation between the more closely packed B850 pigments is such (≈9 Å) that they are excitonically coupled. The structure of the LH2 complex leads to EET from the B800 chromophores to the B850 excitonic states within ~1 ps^3^,^4^,^7^,^8^.

**Figure 4:**
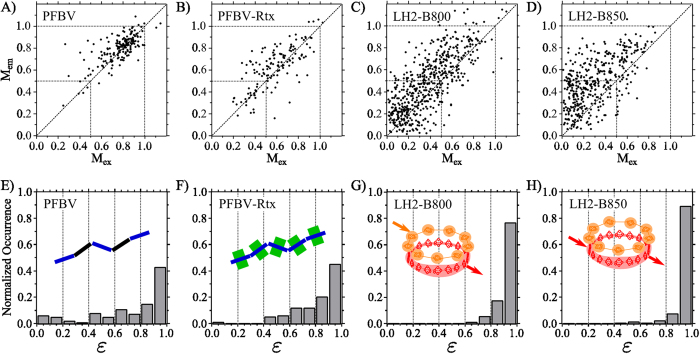
Excitation energy transfer characterization. Modulation depth in excitation and emission correlation plots (**A–D**)^37^,^39^ and funneling efficiency (ε) distribution histograms (**E–H**) for each studied antenna. For the LH2 complexes, excitation wavelength is indicated.

In the LH2-B800 experiment we excited the individual pigments/chromophores of the B800 ring and collected emission from the B850 excitonic states (Fig. 4G). In these conditions, LH2s behave as systems with a single energy funnel: 93% of the complexes could be fitted by the SFA with *ε* > 0.8. This indicates that the energy collected by the B800 chromophores is not only transferred to the B850 ring, but also is always emitted from the same pool of excitonic states of B850 (single EET-emitter).

The B800 to B850 transfer is enhanced by the coupling of the B800 *BChl*s with the nearly resonant B850 exciton levels^44^. The coupling is strengthened by the break-down of the point-dipole approximation for the interaction between the B800 and the excitons of B850 since the latter are spread out over many monomeric units^45^. Recently, coherence in this transfer step has been discussed based on time resolved single LH2 studies^33^.

In the second experiment, 96% of LH2 excited at 850 nm could be fitted by the SFA with *ε* > 0.8, Fig. 4H. This shows that the B850 ring itself behaves as a single funnel. Excitation dynamics in the LH2 B850 ring is mainly determined by the strong interaction between *BChl a* molecules leading to delocalization of the excited states (Frenkel excitons)^46^ over several chromophores. Very ordered systems with high resonance interactions, like organic crystals and J-aggregates, can possess coherent exciton diffusion^47^. However, this mainly occurs at low temperatures^48^. The dynamics in B850 has been well understood in terms of relaxation through the exciton manifold described by Redfield theory or its further modifications^2^. Since different exciton levels reside spatially in different positions of the B850 ring, the relaxation can be visualized as incoherent hopping from one group of the *BChls a* (donor) to another (acceptor)^11^,^13^,^49^. The narrow distribution of *ε* for B850 (Fig. 4H) clearly states that EET between different exciton states is highly efficient and, in equilibrium, the emission is coming from the same pool of states independently of the initially excited ones (perfect energy funneling).

While the overall structure of the LH2 complex is preserved after the spin casting procedure, the 3D orientation of each complex in space is different. These orientational degrees of freedom are the main reason for the large spread of *M*_*ex*_ values observed for the individual LH2 complexes (Fig. 4C,D)^39^,^41^. Moreover, the emission of the B850 ring is in general more polarized than the fluorescence excitation of B800 and B850 (*M*_*em*_ > *M*_*ex*_, Fig. 4C,D). This means that the excitonic states that comprise the EET-emitter of B850 after full relaxation are, to some extent, more localized on a part of the ring than the absorbing states. Consistent with previous observations, this emission localization depends on the particular disorder of the individual LH2 complex, which must be stable over minutes^39^. Further, this indicates that the temperature-induced fluctuations at timescales shorter than 100 s do not completely blur the emission state localization.

### Artificial light-harvesting antennas—CPs

Now the light-harvesting properties of the conjugated polymers PFBV and its analogue with insulated backbone PFBV-Rtx will be discussed. PFBV and PFBV-Rtx chains consist in average of 10 monomeric units (Fig. 1A,B), which translates in an average backbone length of ≈18 nm. The delocalization of the π-electrons in the single chains is disrupted due to energetic and conformational disorder. This leads to the formation of individual chromophores in the conjugated backbone (spectroscopic units)^50^. These energetically similar chromophores are responsible for the observed absorption and emission features. Based on the typical conjugation length of CPs^31^,^37^, we expect PFBV and PFBV-Rtx to have in average approximately five chromophores. After spin coating, the macroscopic shape of each individual chain is different. Therefore, the position and relative orientation of the chromophores varies from chain to chain, resulting in the large spread of *M*_ex_ values observed for both CPs (Fig. 4A,B).

The funneling efficiency in PFBV-Rtx chains (Fig. 4F) is larger than in PFBV chains (Fig. 4E). The main difference between the studied CPs is the presence of the insulating cyclodextrin groups around the PFBV-Rtx backbone (Fig. 1A,B). Therefore, the presence of the insulation somehow improves the ‘communication’ between chromophores in comparison with the ‘bare’ chain.

Previous single molecule fluorescence brightness studies have shown that the majority of the PFBV-Rtx chromophores actively contribute to the fluorescence excitation cross section of the chain^51^. On the other hand, the unprotected PFBV chains have about 2–4 times lower brightness due to the presence of static quenching. Further analysis of the differences between the modulation depth correlation plots of both polymers (Fig. 4A,B) demonstrated that the static quenching in PFBV is inhomogeneous through the backbone^37^. These studies concluded that around 50% of the PFBV chromophores are dark and do not contribute to the fluorescence excitation cross section. Interaction with the environment and peculiarities of chain conformation were suggested as possible reasons of this effect.

In this work we suggest that the reason behind the different energy funneling efficiencies of PFBV and PFBV-Rtx is the difference in the number of active chromophores between these two polymers. When all chromophores of a CP chain are ‘active’ the excitation energy can migrate both through long-range interactions (dipole-dipole-like) and through electronic wave function overlapping (tunneling) of  neighboring excited states. A ‘dark’ chromophore can be seen as e.g. a chain segment with completely different energy level than the rest of the chain. If such segment is present, it works as a spacer increasing the distances between donors and acceptors, and removing the tunneling transfer mechanism. Further, for a smaller number of chromophores, such as in PFBV, there is a larger number of spatial arrangements (orientation factor κ between chromophores) and energetic disorder realization (Förster spectral overlap) for which chromophores cannot efficiently exchange energy^52^,^53^. Obviously these effects hinder EET by making communication between the separated parts of the chain more difficult. Therefore, it is feasible that in PFBV there is a larger number of chain conformations that render the EET process inefficient due to the dipole moment orientation factor and the distances between chromophores in comparison to PFBV-Rtx where all chromophores are active.

There has been a different approach to study EET in isolated CPs based on the observed modulation depth of excitation, emission and the phase shifts^29^. In this approach the conformation of the polymer chains is calculated using Monte Carlo simulations, and then the EET within these conformations is calculated based on Förster theory. This approach potentially can give a full picture of the EET process. However, it relies on knowledge of the spatial arrangement of all chromophores, their energy levels, interactions and mechanisms of the energy transfer. Unfortunately, this microscopic information cannot be directly obtained through experiments. Here the SFA has a great advantage – we do not need to know the details about the object organization and energy transfer mechanisms beforehand. We just check if the EET happens in a directional way or not using only directly observable parameters.

### Comparison between artificial and natural antennas

From the *ε* histograms (Fig. 4 E-H), it is clear that the natural antenna LH2 is by far a better light harvesting system than PFBV and PFBV-Rtx. This occurs despite the similar dimensions of all antenna systems: LH2 diameter is ≈6 nm^43^ (rim length ≈ 19 nm) and the CPs are ≈18 nm long (Fig. 1). On the other hand, the B800 ring has a larger amount of chromophores than the CPs, while the later have larger excitation cross section (≈2 times) per chromophore. This translates into similar absorption capabilities for the B800 ring and the studied polymer molecules.

Note that the modulation correlation plot for PFBV-Rtx and LH2 (Fig. 4B-D) are not fundamentally different. Both systems possess a large spread of the points mostly above the diagonal, indicating the presence of EET towards a more anisotropic group of emissive states. This exemplifies that analyzing the relationship between *M*_*ex*_ and *M*_*em*_ alone is not sufficient to characterize the EET properties of light-harvesting antennas.

The distribution of funneling efficiencies for PFBV-Rtx is quite broad with about 63% of chains possessing *ε* > 0.8 (compared to 95% for LH2) while almost no chains have *ε* < 0.5. Even larger variation was observed for the non-insulated PFBV where a substantial fraction of the chains possessed negligible funneling efficiency (e.g. behaved as set of independent chromophores). The funneling efficiencies of PFBV-Rtx suggests that having closely packed chromophores with similar energies and large dipole moments does not lead in all cases to efficient energy funneling. The variations must be caused by spatial conformation and energetic disorder. In this case, contrary to LH2, the energy absorbed by different dipoles is not able to travel towards a common set of emission dipoles. Admittedly, the presence of individual polymers with large *ε* values shows that certain chain configurations lead to excellent energy funneling properties in these polymers.

Similar to CPs, each LH2 possesses random static disorder, which does not only arises from the random energetic differences between chromophores, but can also be the result of a distortion of the whole complex^26^,^40^,^54^,^55^. This disorder leads to the large spread of values away from the diagonal in the modulation correlation plot of the LH2 complexes, at both B800 and B850 excitation (Fig. 4C,D)^39^. It also reveals itself as variations of emission and excitation spectra of individual LH2s observed at low temperature^56^,^57^,^58^. Contrary to all mentioned properties, the funneling efficiency *ε* is always very close to unity. Therefore, contrasting CPs, the light-harvesting properties of the LH2 complex are remarkably independent of the disorder.

This stability of the light-harvesting properties is the biggest difference between the artificial and natural antennas studied. We attribute this difference to two factors: the spatial arrangement of the chromophores and the nature of the states that exchange energy.

Indeed, the cyclodextrin insulation of the conjugated backbone benefits the intra-EET by removing quenching processes as demonstrated by the comparison between PFBV and PFBV-Rtx^37^,^51^. However, the sophisticated machinery of the LH2 complex does not only protect the pigments from the environment (as cyclodextrin does for the polymer), but also keeps them at a particularly good spatial arrangement. This combination makes the LH2 complex a more robust light-harvesting antenna than the cyclodextrin insulated CP, PFBV-Rtx. Further, the EET in the LH2 complex occurs among partially delocalized exciton states. The energy exchange between these states can be facilitated by higher excitonic states that carry small dipole moment but are delocalized over larger segments of the ring, see ref. 48 and references therein. Such large density of states is not present in the case of the artificial antennas PFBV and PFBV-Rtx due to smaller inter-chromophoric coupling. These factors highlight the importance of structure optimization, in terms of the number and spatial arrangement of chromophores, for the production of efficient artificial light harvesting systems.

## Conclusions

We showed that the light-harvesting properties of individual natural and artificial antennas of completely different organizations can be characterized quantitatively by a parameter called ‘energy funneling efficiency’. This new method is based on the 2D polarization imaging experiment and data analysis in the framework of the single funnel approximation.

This method, specially designed to assess homo-EET, is a further development of fluorescence anisotropy and linear dichroism concepts at the single molecule level. It successfully passed the benchmark test on individual LH2 complexes. These natural antennas possessed a funneling efficiency equal to unity.

Measurements on single conjugated polymers clearly demonstrated that just placing several chromophores with large dipole moment in a close proximity is not enough to ensure efficient energy funneling such as in LH2. Optimization of the spatial arrangement and absence of quenched chromophores is crucial for efficient energy funneling. We believe that conjugated polymer chains as antennas can be improved by combination of the conjugated backbone isolation from the environment (to avoid quenching) with robust structural stability of the optimal spatial configuration, as the Nature does by placing *BChl* molecules in the protein scaffold of LH2.

The main advantage of our method is that it does not require any prior knowledge of the antenna’s organization. Therefore we expect it to have a wide-ranging impact on the study and design of light-harvesting antennas for photovoltaics and artificial photosynthesis.

## Methods

### Sample preparation

#### Single molecules samples of conjugated polymers

Nonpolar butanoylated polyrotaxane polyfluorene bis-vinylphenylene insulated by cyclodextrin (PFBV-Rtx) and its non-insulated counterpart PFBV were synthesized according to the previously published procedure (Fig. 1A,B)^59^. These CPs were both on average 18 nm long (10 monomer units). PFBV-Rtx possessed a threading ratio: 0.8 cyclodextrin per distyrylbenzene unit, which corresponds to a high level of insulation. The preparation of single molecule samples of both CPs was reported before^37^. PFBV and PFBV-Rtx single chains were dispersed onto quartz substrates using polymethyl-methacrylate (PMMA) as polymer matrix.

#### Single molecules samples of LH2

The bacterial light-harvesting complex LH2 (Fig. 1C) of *Rps. acidophila* strain 10050 was isolated as reported elsewhere^60^. The preparation of isolated LH2 complexes on a glass coverslip has been described previously^41^. A solution containing LH2 complexes was deoxygenated by adding an oxygen scavenging system, mixed with polyvinyl alcohol (PVA), and dispersed onto a glass coverslip.

### Experimental setup

Single molecules/complexes were studied using a home-built wide-field fluorescence microscope based on an Olympus IX-71 inverted microscope. For two dimensional polarization imaging the microscope is equipped with an excitation polarization control unit and an emission polarization analyzer as reported before^27^,^30^. Isolated PFBV and PFBV-Rtx were excited using the 458 nm output of an Ar-ion laser; LH2 complexes were excited at 800 nm and 850 nm by a CW Ti:Sapphire laser (Fig. 1). In all cases the excitation light was filtered by a suitable band-pass filter. Isolated CPs and complexes were measured over 1–2 minutes with an average excitation power density of 7 Wcm^−2^ and 2 kWcm^−2^, respectively. All 2D-POLIM measurements were done under an oxygen free atmosphere (nitrogen), see refs 37,39 and Supplementary Information for more details.

## Additional Information

**How to cite this article**: Camacho, R. *et al.* Fluorescence polarization measures energy funneling in single light-harvesting antennas - LH2 vs conjugated polymers. *Sci. Rep.* **5**, 15080; doi: 10.1038/srep15080 (2015).

## Supplementary Material

Supplementary Information

## References

[b1] Van Grondelle R., Dekker J. P., Gillbro T. & Sundstrom V. Energy transfer and trapping in photosynthesis. Biochim. Biophys. Acta 1187, 1–65 (1994).

[b2] Van Grondelle R. & Novoderezhkin V. I. Energy transfer in photosynthesis: experimental insights and quantitative models. Phys. Chem. Chem. Phys. 8, 793–807 (2006). 16482320 10.1039/b514032c

[b3] Freiberg A., Godik V. I., Pullerits T. & Timpman K. Picosecond dynamics of directed excitation transfer in spectrally heterogeneous light-harvesting antenna of purple bacteria. Biochim. Biophys. Acta 973, 93–104 (1989).

[b4] Shreve A. P., Trautman J. K., Frank H. A., Owens T. G. & Albrecht A. C. Femtosecond energy-transfer processes in the B800–850 light-harvesting complex of Rhodobacter sphaeroides 2.4.1. Biochim. Biophys. Acta 1058, 280–288 (1991). 2049375 10.1016/s0005-2728(05)80248-8

[b5] Hess S. *et al.* Femtosecond energy transfer within the LH2 peripheral antenna of the photosynthetic purple bacteria Rhodobacter sphaeroides and Rhodopseudomonas palustris LL. Chem. Phys. Lett. 216, 247–257 (1993).

[b6] Jimenez R., Dikshit S. N., Bradforth S. E. & Fleming G. R. Electronic Excitation Transfer in the LH2 Complex of Rhodobacter sphaeroides. J. Phys. Chem. 100, 6825–6834 (1996).

[b7] Pullerits T., Hess S., Herek J. L. & Sundström V. Temperature Dependence of Excitation Transfer in LH2 of Rhodobacter sphaeroides. J. Phys. Chem. B 101, 10560–10567 (1997).

[b8] Herek J. L. *et al.* B800—>B850 energy transfer mechanism in bacterial LH2 complexes investigated by B800 pigment exchange. Biophys. J. 78, 2590–6 (2000). 10777755 10.1016/S0006-3495(00)76803-2 PMC1300848

[b9] Psencík J., Ma Y.-Z., Arellano J. B., Hála J. & Gillbro T. Excitation energy transfer dynamics and excited-state structure in chlorosomes of Chlorobium phaeobacteroides. Biophys. J. 84, 1161–79 (2003). 12547796 10.1016/S0006-3495(03)74931-5 PMC1302692

[b10] Dostál J. *et al.* Two-dimensional electronic spectroscopy reveals ultrafast energy diffusion in chlorosomes. J. Am. Chem. Soc. 134, 11611–7 (2012). 22690836 10.1021/ja3025627

[b11] Pullerits T., Chachisvilis M. & Sundström V. Exciton Delocalization Length in the B850 Antenna of Rhodobacter sphaeroides. J. Phys. Chem. 100, 10787–10792 (1996).

[b12] Grage M. M.-L. *et al.* Conformational disorder of a substituted polythiophene in solution revealed by excitation transfer. Chem. Phys. Lett. 339, 96–102 (2001).

[b13] Olaya-Castro A. & Scholes G. D. Energy transfer from Förster–Dexter theory to quantum coherent light-harvesting. Int. Rev. Phys. Chem. 30, 49–77 (2011).

[b14] Olbrich C. & Kleinekathöfer U. Time-dependent atomistic view on the electronic relaxation in light-harvesting system II. J. Phys. Chem. B 114, 12427–37 (2010). 20809619 10.1021/jp106542v

[b15] Paillotin G., Swenberg C. E., Breton J. & Geacintov N. E. Analysis of picosecond laser induced fluorescence phenomena in photosynthetic membranes utilizing a master equation approach. Biophys. J. 25, 513–33 (1979). 262402 10.1016/S0006-3495(79)85320-5 PMC1328488

[b16] Valkunas L., Trinkunas G., Liuolia V. & van Grondelle R. Nonlinear annihilation of excitations in photosynthetic systems. Biophys. J. 69, 1117–29 (1995). 8519966 10.1016/S0006-3495(95)79986-6 PMC1236340

[b17] Scheblykin I. G. *et al.* Non-coherent exciton migration in J-aggregates of the dye THIATS: exciton–exciton annihilation and fluorescence depolarization. Chem. Phys. Lett. 298, 341–350 (1998).

[b18] Balaban T. S. Tailoring porphyrins and chlorins for self-assembly in biomimetic artificial antenna systems. Acc. Chem. Res. 38, 612–23 (2005). 16104684 10.1021/ar040211z

[b19] Röger C. *et al.* Efficient energy transfer from peripheral chromophores to the self-assembled zinc chlorin rod antenna: a bioinspired light-harvesting system to bridge the ‘green gap’. J. Am. Chem. Soc. 128, 6542–3 (2006). 16704238 10.1021/ja0584469

[b20] Miyatake T. & Tamiaki H. Self-aggregates of natural chlorophylls and their synthetic analogues in aqueous media for making light-harvesting systems. Coord. Chem. Rev. 254, 2593–2602 (2010).

[b21] Chappaz-Gillot C. *et al.* Anisotropic organization and microscopic manipulation of self-assembling synthetic porphyrin microrods that mimic chlorosomes: bacterial light-harvesting systems. J. Am. Chem. Soc. 134, 944–54 (2012). 22148684 10.1021/ja203838p

[b22] Furumaki S., Vacha F., Hirata S. & Vacha M. Bacteriochlorophyll aggregates self-assembled on functionalized gold nanorod cores as mimics of photosynthetic chlorosomal antennae: a single molecule study. ACS Nano 8, 2176–82 (2014). 24559170 10.1021/nn500224v

[b23] Sforazzini G. *et al.* Synthesis and Photophysics of Coaxial Threaded Molecular Wires: Polyrotaxanes with Triarylamine Jackets. J. Phys. Chem. C 118, 4553–4566 (2014).

[b24] Vanden Bout D. A. *et al.* Discrete Intensity Jumps and Intramolecular Electronic Energy Transfer in the Spectroscopy of Single Conjugated Polymer Molecules. Science 277, 1074–1077 (1997).

[b25] Hu D., Yu J. & Barbara P. F. Single-molecule spectroscopy of the conjugated polymer MEH-PPV. J. Am. Chem. Soc. 121, 6936–6937 (1999).

[b26] Cogdell R. J. & Köhler J. Use of single-molecule spectroscopy to tackle fundamental problems in biochemistry: using studies on purple bacterial antenna complexes as an example. Biochem. J. 422, 193–205 (2009). 19663809 10.1042/BJ20090674

[b27] Mirzov O. *et al.* Polarization portraits of single multichromophoric systems: visualizing conformation and energy transfer. Small 5, 1877–88 (2009). 19384881 10.1002/smll.200801168

[b28] Becker K. *et al.* Efficient intramolecular energy transfer in single endcapped conjugated polymer molecules in the absence of appreciable spectral overlap. J. Am. Chem. Soc. 128, 680–1 (2006). 16417332 10.1021/ja056469h

[b29] Traub M. C., Lakhwani G., Bolinger J. C., Vanden Bout D. & Barbara P. F. Electronic energy transfer in highly aligned MEH-PPV single chains. J. Phys. Chem. B 115, 9941–7 (2011). 21812493 10.1021/jp204591p

[b30] Camacho R., Thomsson D., Yadav D. & Scheblykin I. G. Quantitative characterization of light-harvesting efficiency in single molecules and nanoparticles by 2D polarization microscopy: Experimental and theoretical challenges. Chem. Phys. 406, 30–40 (2012).

[b31] Lin H. *et al.* Fluorescence blinking, exciton dynamics, and energy transfer domains in single conjugated polymer chains. J. Am. Chem. Soc. 130, 7042–51 (2008). 18473464 10.1021/ja800153d

[b32] Müller J. G., Lupton J. M., Feldmann J., Lemmer U. & Scherf U. Ultrafast intramolecular energy transfer in single conjugated polymer chains probed by polarized single chromophore spectroscopy. Appl. Phys. Lett. 84, 1183 (2004).

[b33] Hildner R., Brinks D., Nieder J. B., Cogdell R. J. & Van Hulst N. F. Quantum coherent energy transfer over varying pathways in single light-harvesting complexes. Science 340, 1448–51 (2013). 23788794 10.1126/science.1235820

[b34] Merdasa A. *et al.* Single Lévy States-Disorder Induced Energy Funnels in Molecular Aggregates. Nano Lett. 14, 6774–6781 (2014). 25349900 10.1021/nl5021188

[b35] Lin H. *et al.* Single chain versus single aggregate spectroscopy of conjugated polymers. Where is the border? Phys. Chem. Chem. Phys. 12, 11770–7 (2010). 20694242 10.1039/c001120g

[b36] Bounos G. *et al.* Controlling chain conformation in conjugated polymers using defect inclusion strategies. J. Am. Chem. Soc. 133, 10155–60 (2011). 21612291 10.1021/ja2006687

[b37] Camacho R., Thomsson D., Sforazzini G., Anderson H. L. & Scheblykin I. G. Inhomogeneous Quenching as a Limit of the Correlation Between Fluorescence Polarization and Conformation of Single Molecules. J. Phys. Chem. Lett. 4, 1053–1058 (2013). 26291377 10.1021/jz400142x

[b38] Tian Y. *et al.* Organization of bacteriochlorophylls in individual chlorosomes from Chlorobaculum tepidum studied by 2-dimensional polarization fluorescence microscopy. J. Am. Chem. Soc. 133, 17192–9 (2011). 21923120 10.1021/ja2019959

[b39] Tubasum S. *et al.* Evidence of excited state localization and static disorder in LH2 investigated by 2D-polarization single-molecule imaging at room temperature. Phys. Chem. Chem. Phys. 15, 19862–9 (2013). 24145962 10.1039/c3cp52127c

[b40] Bopp M. A., Sytnik A., Howard T. D., Cogdell R. J. & Hochstrasser R. M. The dynamics of structural deformations of immobilized single light-harvesting complexes. PNAS 96, 11271–11276 (1999). 10500166 10.1073/pnas.96.20.11271 PMC18023

[b41] Tubasum S., Cogdell R. J., Scheblykin I. G. & Pullerits T. Excitation-emission polarization spectroscopy of single light harvesting complexes. J. Phys. Chem. B 115, 4963–70 (2011). 21486039 10.1021/jp107480x

[b42] Camacho R. *et al.* Polarization imaging of emissive charge transfer states in polymer/fullerene blends. Chem. Mater. 26, 6695–6704 (2014).

[b43] McDermott G. *et al.* Crystal structure of an integral membrane light-harvesting complex from photosynthetic bacteria. Nature 374, 517–521 (1995).

[b44] Scholes G. D. & Fleming G. R. On the Mechanism of Light Harvesting in Photosynthetic Purple Bacteria: B800 to B850 Energy Transfer. J. Phys. Chem. B 104, 1854–1868 (2000).

[b45] Beenken W. J. D. & Pullerits T. Excitonic coupling in polythiophenes: comparison of different calculation methods. J. Chem. Phys. 120, 2490–5 (2004). 15268391 10.1063/1.1636460

[b46] Kasha M., Rawls H. R. & Ashraf El-Bayoumi M. The exciton model in molecular spectroscopy. Pure Appl. Chem. 11, 371–392 (1965).

[b47] Pope M. & Swenberg C. E. Electronic Processes in Organic Solids. Annu. Rev. Phys. Chem. 35, 613–655 (1984).

[b48] Scheblykin I. G. In J-Aggregates Volume 2 (ed. Kobayashi T.) 247–271 (World Scientific, 2012).

[b49] Sumi H. Theory on Rates of Excitation-Energy Transfer between Molecular Aggregates through Distributed Transition Dipoles with Application to the Antenna System in Bacterial Photosynthesis. J. Phys. Chem. B 103, 252–260 (1999).

[b50] Beenken W. J. D. & Pullerits T. Spectroscopic units in conjugated polymers: a quantum chemically founded concept? J. Phys. Chem. B 108, 6164–9 (2004). 18950096 10.1021/jp037332l

[b51] Thomsson D. *et al.* Cyclodextrin insulation prevents static quenching of conjugated polymer fluorescence at the single molecule level. Small 9, 2619–27 (2013). 23463732 10.1002/smll.201203272

[b52] Ford J. S. & Andrews D. L. Geometrical effects on resonance energy transfer between orthogonally-oriented chromophores, mediated by a nearby polarisable molecule. Chem. Phys. Lett. 591, 88–92 (2014).

[b53] Buckhout-White S. *et al.* Assembling programmable FRET-based photonic networks using designer DNA scaffolds. Nat. Commun. 5, 5615 (2014). 25504073 10.1038/ncomms6615 PMC4275599

[b54] Van Oijen A. M., Ketelaars M., Köhler J., Aartsma T. J. & Schmidt J. Unraveling the Electronic Structure of Individual Photosynthetic Pigment-Protein Complexes. Science 285, 400–402 (1999). 10411501 10.1126/science.285.5426.400

[b55] Hong X., Weng Y.-X. & Li M. Determination of the topological shape of integral membrane protein light-harvesting complex LH2 from photosynthetic bacteria in the detergent solution by small-angle X-ray scattering. Biophys. J. 86, 1082–8 (2004). 14747343 10.1016/S0006-3495(04)74183-1 PMC1303901

[b56] Matsushita M. *et al.* Spectroscopy on the B850 band of individual light-harvesting 2 complexes of Rhodopseudomonas acidophila. II. Exciton states of an elliptically deformed ring aggregate. Biophys. J. 80, 1604–14 (2001). 11222321 10.1016/S0006-3495(01)76133-4 PMC1301352

[b57] Novoderezhkin V. I., Rutkauskas D. & van Grondelle R. Dynamics of the emission spectrum of a single LH2 complex: interplay of slow and fast nuclear motions. Biophys. J. 90, 2890–902 (2006). 16443651 10.1529/biophysj.105.072652 PMC1414546

[b58] Kunz R. *et al.* Single-molecule spectroscopy unmasks the lowest exciton state of the B850 assembly in LH2 from Rps. acidophila. Biophys. J. 106, 2008–2016 (2014). 24806933 10.1016/j.bpj.2014.03.023 PMC4017283

[b59] Brovelli S. *et al.* Emission Color Trajectory and White Electroluminescence Through Supramolecular Control of Energy Transfer and Exciplex Formation in Binary Blends of Conjugated Polyrotaxanes. Adv. Funct. Mater. 22, 4284–4291 (2012).

[b60] Cogdell R. J., Woolley K. J., Ferguson L. A. & J D. D. Crystallization of purple bacteria antenna complexes. (CRC Press, 1991).

