# Supplementary material for: Fluorescence polarization measures energy funneling in single light-harvesting antennas—LH2 vs conjugated polymers
Source: Sci Rep. 2015 Oct 19;5:15080. doi: 10.1038/srep15080 (PMC4609963; doi:10.1038/srep15080)
Supplement: Supplementary Information [file srep15080-s1.pdf]

Supplementary Information
Fluorescence polarization measures energy funneling in single light-harvesting antennas – LH2 vs conjugated polymers

Rafael Camacho,† Sumera Tubasum,† June Southall,‡ Richard J. Cogdell,‡ Giuseppe Sforazzini,§ Harry L. Anderson,§ Tõnu Pullerits† and Ivan G. Scheblykin†*

† Chemical Physics, Lund University, PO Box 124, Lund, SE-22100, Sweden

‡ Glasgow Biomedical Research Centre, University of Glasgow, G12 8QQ, United Kingdom

§ Department of Chemistry, University of Oxford, Mansfield Road, Oxford, OX1 3TA, United Kingdom

* Corresponding author E-mail: Ivan.Scheblykin@chemphys.lu.se

Detailed description of the experimental setup for two-dimensional polarization imaging, 2D-POLIM

2D POLIM experiments were performed using a home-built wide-field fluorescence microscope based on a commercial Olympus IX71 inverted microscope. The conjugated polymers (CP) were excited using the 458 nm output of an Ar-Ion laser, while the LH2 were excited at 800 nm and 850 nm by a tunable CW Ti:Sapphire laser. The laser light was passed through a suitable clean-up filter before reaching the sample plane. The excitation controller was used to change the orientation of the linearly polarized light (θ_{ex}) on the sample plane and consisted of an appropriate λ/2 achromatic plate (Thorlabs) mounted in a motorized rotation mount (home-built). Some of the optical elements placed between the excitation controller and the sample plane are birefringent and thus change the polarization state of the initially linearly polarized excitation light at the sample plane. Therefore, a Berek compensator (New Focus) was placed after the excitation controller to introduce the opposite phase shift and recover the linear polarization at the sample plane. A defocusing wide-field lens was used to obtain an excitation spot with diameter of ≈30 μm at the sample plane. An oil immersion objective lens (60x Olympus, UPlanFLN, NA = 0.65 for CPs and NA = 1.25 for LH2) was used to image the sample. The de-collimation of the beam, together with a small fill-factor at the objective's back aperture, led to a numerical aperture during excitation less than 0.15. The samples were kept under nitrogen to avoid photo-induced oxidation.

The fluorescence was collected with the same objective lens and leaved the microscope through a special port, which used a mirror instead of a prism to avoid polarization artifacts in emission. Appropriate emission filters (two filters together) were used to select the spectral range of the fluorescence to be detected. For CPs: 465 and 470 nm long pass filters (Chroma). For LH2: (i) 840 and 830 nm long pass filters (Chroma) for 800 nm excitation; (ii) 870 and 860 nm long pass filters (Chroma) for 850 nm excitation. The emission analyzer was used to control the emission polarization orientation (θ_{em}). It consisted of a wire-grid linear polarizer (Edmund optics for CPs and VLS-100-NIR, Meadow- lark optics for LH2) mounted in a motorized rotating mount (home-built). Finally, the fluorescence emission was imaged on an EMCCD camera (Princeton Instruments, PhotonMax 512).

Both rotating mounts were controlled by stepper motors and continuously rotated while the sample was imaged. To probe many different angle combinations (φ_{ex}, φ_{em}) the rotation frequencies of both motors were different. The total magnification of the optical system was about 200 times.

For the study of LH2 a dichroic mirror could not be used due to its extremely large birefringence at wavelengths near the IR spectral region. To solve this problem, we used a so-called polarization preserving universal beam splitter (Figure S1-B), which replaces the dichroic mirror in the standard filter cube. The universal beam splitter consists of a glass plate with anti-reflection coatings on both sides and very small metal mirror in the middle (diameter ≈ 1 mm). The excitation light is focused on the plane of the small mirror by a

carefully chosen wide-field lens (WFL, Figure S1-B), to a diameter small enough to be fully reflected by the small mirror. The focal distance of the WFL is selected in such a way that the excitation light is focused by the microscope objective lens above its focal distance, and generates wide-field illumination of the sample. The fluorescence collected by the microscope objective results in a beam of much larger diameter than the small mirror. Therefore, the light obstruction by the mirror is negligible. The birefringence of the universal beam splitter proved to be much smaller than that of the dichroic mirror for the near IR light.

For more details about 2D-POLIM and experimental artifacts in polarization sensitive fluorescence microscopies see Camacho, R. Polarization Portraits of Light-Harvesting Antennas: From Single Molecule Spectroscopy to Imaging, Lund University, 2014, p. 202. DOI: 10.13140/2.1.4852.5607.

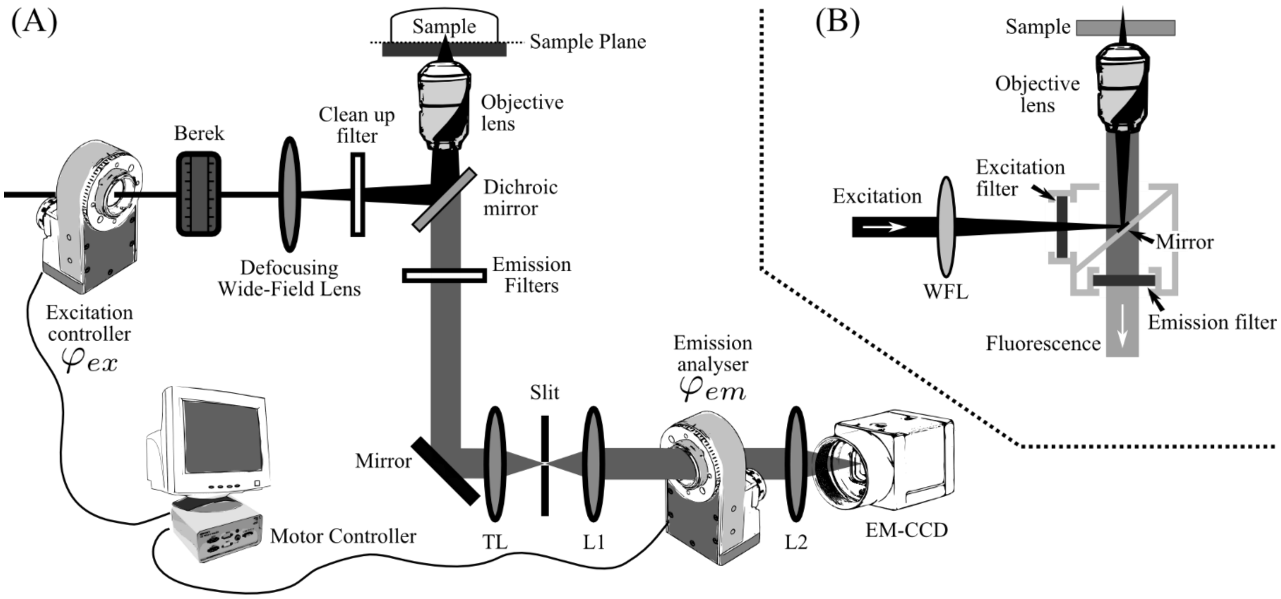

Figure S1: Experimental setup for 2D-POLIM. (A) Schematics of the experimental setup. TL: tube lens. L1, L2 - lens. Rotation of the excitation controller and emission analyzer was done by stepper motors. The excitation controller defines the excitation polarization angle at the sample plane, φ_{ex}. The emission analyzer defines the emission polarization angle, φ_{em}. (B) Polarization preserving universal beam splitter. This device is used when the birefringence of the dichroic mirror is so large that it cannot be used for polarization sensitive measurements.

Reasoning behind the approximations done in the Single Funnel Approximation

In order to extract information from a polarization portrait, which only has 9 degrees of freedom, when the organization and detailed spectroscopic information of each transition in the multichromophoric system is unknown, several approximations have to be made. As mentioned in the main text these approximations are: (1) the EET drives the excitations towards a fixed set of chromophores called EET-emitter (a common pool of emitting states), (2) the unknown N-dipole system is represented by a new group of Ñ standard dipoles with equal fluorescence excitation cross sections ($\sigma^{ex} \sim \sigma \times \Phi$), and (3) each dipole transfers in average the same amount of energy towards the single EET-emitter.

Approximation 1) means that the SFA does not consider time changes in the transfer efficiency between chromophores, and that the experiment is done under steady state conditions. Approximation 2) is a change in coordinate system. As a consequence of this change, the SFA cannot keep track of how many excitations get lost through non-radiative decays for each chromophore. Finally, approximation 3), which can be seen as the biggest approximation of the SFA, is used because this is exactly the property that the SFA is designed to measure (the ability of a system to directionally transfer their energy to a specific set of dipoles). In case that approximation 3) does not hold for the measured multichromophoric system, then the SFA will measure a low funneling efficiency or will not be able to fit the data, which is an acceptable result for the SFA.

Description of the Single Funnel Approximation fitting procedure

A detailed description about the single funnel approximation and the equations used for data fitting can be found in reference 30 of the main text and Camacho, R. Polarization Portraits of Light-Harvesting Antennas: From Single Molecule Spectroscopy to Imaging, Lund University, 2014, p. 202. DOI: 10.13140/2.1.4852.5607. In the following section we briefly describe the equations used for data fitting.

As mentioned in the main text, the polarization portrait, $I(\varphi_{ex}, \varphi_{em})$, can be split into two components using the single funnel approximation (SFA):

$$I(\varphi_{ex}, \varphi_{em}) \approx (1-\varepsilon)A + \varepsilon B \quad (2)$$

$$A = \tilde{N} \sum_{i=1}^{\tilde{N}} \cos^2(\varphi_{ex} - \alpha_i) \cos^2(\varphi_{em} - \alpha_i) \quad (3)$$

$$B = \frac{\tilde{N}}{2}\left[1 + M_f \cos\left(2\left[\varphi_{em} - \theta_f\right]\right)\right] \sum_{i=1}^{\tilde{N}} \cos^2(\varphi_{ex} - \alpha_i) \quad (4)$$

where A and B describe the behavior of an independent group of chromophores and a single EET-emitter, respectively. $\tilde{N}$ is the number of standard dipoles (having equal fluorescence excitation cross sections) used to represent the unknown experimental system of N dipoles. φ_{ex} and φ_{em} are the excitation polarization plane orientation and the orientation of the emission analyzer, respectively. α_i is the in plane orientation of the standard dipole 'i'. M_f and θ_f are the polarization degree and main orientation of the EET-emitter, respectively.

In order to use equations 2-4 to fit a polarization portrait we have to define a methodology to find the number and orientation of standard dipoles to be used. The methodology we have chosen is based in a symmetric three-dipole-model.[27, 30 from main text] In this approach, the system of $\tilde{N}$ dipoles with identical fluorescence excitation cross section (σ^{ex}) is represented by three dipoles with the following properties:

1) A main dipole (i=1), which defines the main orientation of the system and has orientation α_1 and fluorescence excitation cross section σ^{ex}_1.
2) Two side dipoles (i=2 and 3) of identical fluorescence excitation cross section ($\sigma^{ex}_2 = \sigma^{ex}_3$) and symmetrically oriented around the main dipole ($\alpha_2 = \alpha_1 + \delta$, $\alpha_3 = \alpha_1 - \delta$).

Such three dipole model possesses the following degrees of freedom: orientation of the main dipole (α_1), orientation of the side dipoles respective to the main dipole (δ) and the ratio between the excitation cross section of the main and side dipoles ($\Gamma = \sigma_1^{ex}/\sigma_2^{ex} = \sigma_1^{ex}/\sigma_3^{ex}$). Note that this three-dipole-model is identical to a systems composed of $c(\Gamma + 2)$ dipoles of identical σ^{ex}, where:

i) $(c \times \Gamma)$ dipoles are oriented along α_1.
ii) c dipoles are oriented along α_2
iii) c dipoles oriented along α_3
iv) c is a factor to make Γ an integer.

Moreover, the use of the symmetric three-dipole-model is preferred because it simplifies the equations used for data fitting.

Using the symmetric three-dipole-model equations 3 takes the form:

$$\begin{aligned} A = [&\Gamma \cos^2(\varphi_{ex} - \theta_{ex}) \cos^2(\varphi_{em} - \theta_{ex}) \\ &+ \cos^2(\varphi_{ex} - [\theta_{ex} + \delta]) \cos^2(\varphi_{em} - [\theta_{ex} + \delta]) \\ &+ \cos^2(\varphi_{ex} - [\theta_{ex} - \delta]) \cos^2(\varphi_{em} - [\theta_{ex} - \delta])] \end{aligned} \quad (S01)$$

In equation S01, $\alpha_1 \equiv \theta_{ex}$, where θ_{ex} is the experimentally determined fluorescence excitation polarization phase. This is because the orientation of the main dipole in the symmetric three-dipole-model must be equal to the main orientation axis of the transition dipoles responsible for fluorescence excitation.

Further, it can be shown that the last summation of equation 4 can be written as:

$$\sum_{i=1}^{\tilde{N}} \tilde{N} \cos^2(\varphi_{ex} - \alpha_i) = \mathrm{K} \cos^2(\omega - \theta) + \mathrm{M} = \frac{\tilde{N}}{2}[1 + M_{ex} \cos(2[\varphi_{ex} - \theta_{ex}])] \qquad (S02)$$

where M_{ex} is the experimentally determined fluorescence excitation modulation depth.

Using S02 and after a normalization step ($\tilde{N} = 2 + \Gamma$), equation 3 and 4 can be written as:

$$A = \frac{1}{2 + \Gamma}[\Gamma \cos^2(\varphi_{ex} - \theta_{ex}) \cos^2(\varphi_{em} - \theta_{ex}) + \cos^2(\varphi_{ex} - [\theta_{ex} + \delta]) \cos^2(\varphi_{em} - [\theta_{ex} + \delta]) + \cos^2(\varphi_{ex} - [\theta_{ex} - \delta]) \cos^2(\varphi_{em} - [\theta_{ex} - \delta])] \qquad (S03)$$

$$B = \frac{1}{4}[1 + M_{ex} \cos(2[\varphi_{ex} - \theta_{ex}])][1 + M_f \cos(2[\varphi_{em} - \theta_f])] \qquad (S04)$$

where δ is given by:

$$\delta(\Gamma, M_{ex}) = \frac{1}{2} \arccos\left[\frac{(\Gamma + 2)M_{ex} - \Gamma}{2}\right] \qquad (S05)$$

Equations S03 and S04 are used for fitting experimentally determined polarization portraits, $I(\varphi_{ex}, \varphi_{em})$. During the fitting procedure 4 parameters are changed: Γ, M_f, θ_f, and ε. Moreover, these parameter are bounded in the following way:

$$0 \leq \varepsilon \leq 1 \qquad (S06)$$

$$0 \leq M_f \leq 1 \qquad (S07)$$

$$-90 < \theta_f \leq 90 \quad [degrees] \qquad (S08)$$

$$0 \leq \Gamma \leq 2\frac{1 + M_{ex}}{1 - M_{ex}} \qquad (S09)$$

Note that equation S09, and as a consequence S05, are not defined for $M_{ex} = 1$. Therefore, a special case is defined for multichromophoric systems with $M_{ex} = 1$. In this special case the absorbing dipoles are modelled by a single dipole oriented at $\alpha = \theta_{ex}$ (instead of the symmetric three-dipole-model), and equations S03 and S04 take the form:

$$A = \cos^2(\varphi_{ex} - \theta_{ex}) \cos^2(\varphi_{em} - \theta_{ex}) \qquad (S10)$$

$$B = \cos^2(\varphi_{ex} - \theta_{ex}) \frac{1}{2}[1 + M_f \cos(2[\varphi_{em} - \theta_f])] \qquad (S11)$$
